# Probing Public Perceptions of Antidepressants on Social Media: Mixed Methods Study

**DOI:** 10.2196/62680

**Published:** 2025-02-26

**Authors:** Jianfeng Zhu, Xinyu Zhang, Ruoming Jin, Hailong Jiang, Deric R Kenne

**Affiliations:** 1 Department of Computer Science, Kent State University, 800 E Summit St, Kent, OH, 44242, United States, 1 3306729980; 2 Department of Communication, Michigan State University, East Lansing, MI, United States; 3 Center for Public Policy and Health, College of Public Health, Kent State University, Kent, OH, United States

**Keywords:** antidepressant, AskaPatient, natural language processing, BERTopic, large language models, Reddit

## Abstract

**Background:**

Antidepressants are crucial for managing major depressive disorders; however, nonadherence remains a widespread challenge, driven by concerns over side effects, fear of dependency, and doubts about efficacy. Understanding patients’ experiences is essential for improving patient-centered care and enhancing adherence**,** which prioritizes individual needs in treatment.

**Objective:**

This study aims to gain a deeper understanding of patient experiences with antidepressants, providing insights that health care providers, families, and communities can develop into personalized treatment strategies. By integrating patient-centered care, these processes may improve satisfaction and adherence with antidepressants.

**Methods:**

Data were collected from AskaPatient and Reddit, analyzed using natural language processing and large language models. Analytical techniques included sentiment analysis, emotion detection, personality profiling, and topic modeling. Furthermore, demographic variations in patient experiences were also examined to offer a comprehensive understanding of discussions around antidepressants.

**Results:**

Sentiment and emotion analysis revealed that the majority of discussions (21,499/36,253, 59.3%) expressed neutral sentiments, with negative sentiments following closely (13,922/36,253, 38.4%). The most common emotions were fear (16,196/36,253, 44.66%) and sadness (12,507/36,253, 34.49%). The largest topic, “Mental Health and Relationships,” accounted for 11.69% (3755/36,253) of the discussions, which indicated a significant focus on managing mental health conditions. Discussions around nonadherence were marked by fear, followed by sadness, while self-care discussions showed a notable trend of sadness.

**Conclusions:**

These psychological insights into public perceptions of antidepressants provide a foundation for developing tailored, patient-centered treatment approaches that align with individual needs, enhancing both effectiveness and empathy of care.

## Introduction

### Background

Antidepressants are a cornerstone in the treatment of major depressive disorder worldwide, improving the quality of life for millions of individuals. However, nonadherence to antidepressant therapy remains a significant barrier to achieving successful outcomes. According to the National Center for Health Statistics, 13.2% of adults in the United States used antidepressants within the past 30 days, with higher rates among women (17.7%) than among men (8.4%) between 2015 and 2018 [1]. This disparity highlights potential differences in how men and women experience depression and respond to antidepressant therapy, which may influence their adherence behaviors [2].

The factors driving nonadherence are multifaceted, encompassing both patient- and clinician-related challenges. Patients may avoid medicines due to concerns about side effects, fear of dependency, or doubts about their effectiveness [3]. Furthermore, depression’s symptoms, such as low motivation and emotion dysregulated, often prevent patients from filling prescriptions or attending follow-up appointments [2]. Clinician-related factors, such as insufficient patient education and inadequate follow-up, also contribute to poor adherence. These challenges underscore the need for a more patient-centered approach in managing depression.

Patient-centered care (PCC) is designed to optimize treatment adherence by ensuring that clinical interventions are closely aligned with each patient’s preferences, needs, and values. This approach enhances communication between caregivers and patients, supports collaborative decision-making, and increase overall patient satisfaction, all of which are essential to improving adherence rates [4,5]. However, long-term treatment with selective serotonin reuptake inhibitors raises concerns about emotion blunting, a condition in which patients report feeling emotionally numb, impacting both their positive and negative emotions, which potentially alter personality [6-8]. This highlights the need to examine the emotional and psychological dimensions of antidepressant use in real-world setting.

Traditional methods such as surveys and interviews are limited by recall bias and cannot capture the day-to-day emotional fluctuations that significantly influence medication adherence [9,10]. Real-time insights are crucial to understanding how emotions, which change rapidly in response treatment, impact medication adherence. Given that medication adherence is closely linked to patient emotions during treatment, consequently there is a need for innovative data collection that can offer information on patients’ real-time experience.

Social media offers a unique opportunity to capture real-time, unfiltered opinions from users [11-13]. Unlike structured clinical trials or surveys, social media discussions reflect spontaneous, in the moment experiences, offering deeper insights into how patients perceive their medications and mental health [14-16]. Furthermore, social media provides large-scale, real-time data on patient experiences, which can be analyzed to better understand the emotional and psychological factors influencing antidepressant adherence.

Advancements in artificial intelligence, particularly with natural language processing (NLP) and large language models (LLMs), such as GPT-4, allow for more nuanced analysis of patient language [17-19]. These tools provide deeper insights by not only detecting sentimental analysis but also profiling personality traits, allowing researchers to explore psychological dimensions of antidepressant users. Through NLP and LLMs, it is possible to assess emotional states, sentiment, and personality traits from large volumes of patient-generated content.

This study aims to bridge the gap between clinical assessment and patient-reported experiences by using real-time social media data. This advanced approach offers a more comprehensive understanding of patient experiences, capturing emotional and psychological factors often missed by traditional methods. The findings support decision-making strategies for caregivers and enhance PCC strategies leading to increase treatment adherence.

### Literature Review

#### Nonadherence to Antidepressants

Research consistently highlights significant variability in antidepressant adherence across different populations. A review of studies over the past decade reveals that approximately 50% of patients discontinue antidepressant therapy prematurely, which underscore the pervasive nature of nonadherence [3]. Pompili et al [20] identified several key predictors of nonadherence in patients with mood disorders, including those of younger age (younger than 40 years), comorbid substance use or personality disorders, negative beliefs, poor insight, treatment-related side effects, and a weak therapeutic alliance. The study on 332 patients by Kamaradova et al [21] found a negative correlation between self-stigma and adherence, with greater illness severity associated with lower adherence across all diagnostic groups. Similarly, De las Cuevas et al [22] identified that adherence was closely linked to positive attitudes toward treatment, while nonadherence was associated with severe depression and concerns about side effects. These findings underscore the multifactorial nature of antidepressant nonadherence, influenced by patient beliefs, illness severity, and the quality of patient-provider relationships [23].

#### Patient-Centered Care

PCC, which prioritizes the patient’s preferences and values, plays a crucial role in addressing nonadherence. It improves patient satisfaction and outcomes by considering the psychological challenges, such as fear and uncertainty, faced by patients and their families [24]. Studies show that shared decision-making and effective communication between health care providers and patients foster trust, enabling patients to express their emotional concerns, ultimately strengthening the therapeutic alliance, and improving adherence [25,26].

Integration sentiment analysis into PCC allows health care providers to gain real-time insights into patient emotions and concerns, enabling timely interventions that enhance adherence and promote self-care strategies [27]. These tools, combined with personality profiling, offer a more nuanced understanding of patient experiences than traditional methods [28], allowing for personalized care that improves mental health outcomes.

#### Use of Social Media for Health Research

Social media provides real-time, unfiltered insights into patient experiences with medication and mental health challenges. Platforms such as X (Twitter) and Reddit are rich sources of data that help researchers understand patient interactions with their mental health conditions and the health care system. Social media enables real-time health monitoring and predictive analysis, allowing for early detection of changes in patient mood or attitudes toward medications, which can trigger timely interventions for nonadherence [29-31]. Sentiment analysis of social media posts has proven effective in identifying concerns about side effects, medication efficacy, and treatment challenges, complementing traditional health care systems by offering new avenues for improving adherence to antidepressants [30].

#### Integration of NLP and LLMs in Mental Health Research

Recent NLP models have shown promise in identifying and classifying even rare neuropsychiatric diseases. For instance, the language model RoBERTa has been used to identify clusters of individuals with primary progressive aphasia through speech analysis, providing health care providers with valuable insights into patient experiences [32,33]. LLMs, which increasingly power conversational agents, can simulate synthetic personalities, allowing for fine-tuned, personalized patient interactions. These models amplify mental health support by offering tailored perspectives that improve adherence to therapy and foster more effective patient care [34].

Emotion-based models, such as those using emotional transition fingerprints, have demonstrated greater generalizability than content-based models, helping researchers understand the social dynamics of antidepressant use [35]. Furthermore, BERT has been used to categorize antidepressant-related discussions on social media, such as mood, sleep, and somatic symptoms, showcasing NLP’s ability to provide targeted analysis in specific domains [36]. These tools have also been applied to predict drug interactions and optimize treatment plans using techniques such as Adaptive Fuzzy Logic Neural Networks, improving accuracy in predicting side effects [37].

Despite substantial research on antidepressant nonadherence, existing studies often neglect the dynamic interplay of emotions, personality traits, and social media discourse in shaping patient behaviors. Traditional approaches have provided foundational insights, yet they frequently fail to capture the real-time and nuanced patient experiences accessible through modern social media platforms. Furthermore, while PCC is increasingly recognized for its potential to improve adherence, there is a critical gap on how to leverage advanced NLP and LLMs to investigate patients’ present psychological reaction with antidepressants using social media datasets.

This research aims to bridge the gap, which provides a more granular understanding of the psychological factors by analyzing sentiment, emotion, and personality profiles related to antidepressant use across diverse demographics. Furthermore, we explore topics, especially topics associated with 2 themes: nonadherence and self-care across sentiment and personality profile, which enhances our understanding of antidepressant users’ real-time psychological patterns. The following 4 research questions (RQs) align to the goal of this study.

RQ1: How do sentiment analyses reveal differences in antidepressant experiences across demographics?

RQ2: What predominant emotions (eg, fear, sadness, and joy) are associated with antidepressant use, and how do these vary by demographics?

RQ3: What personality traits (eg, extraversion and neuroticism) are prevalent among users discussing antidepressants, and how do these traits differ across demographics?

RQ4: What are the prevailing topics in web-based antidepressant discussions, and how do sentiment, emotion, and personality insights inform our understanding of nonadherence and self-care themes?

Through these questions, this study addresses key limitations identified in the existing literature and harnesses advanced NLP techniques on social media data. The findings offer deeper understanding of patients’ experience, improving antidepressant treatments.

## Methods

### Data Collection and Preprocessing for Antidepressant Analysis

To explore public perceptions of antidepressants, data were collected from 2 major platforms: AskaPatient [38] and Reddit [39], both of which offer diverse, patient-reported experiences. AskaPatient provides structured reviews and ratings, while Reddit features spontaneous, unfiltered discussions, offering a broader range of insights.

From AskaPatient, data were collected for the top 10 antidepressants, based on 1571 reviews posted between 2015 and 2023. This dataset includes demographic information (eg, gender and age), satisfaction ratings, side effects, and free-text comments (see Table 1 for sample data). The analyzed antidepressants were Cymbalta, Effexor XR, Lexapro, Zoloft, Wellbutrin XL, Wellbutrin, Celexa, Paxil, Wellbutrin SR, and Prozac.

We filtered posts from Reddit between 2018 and 2023 using the same top 10 antidepressants analyzed in the AskaPatient dataset. Due to the absence of explicit demographic details in Reddit posts, we used regular expressions (regex) to deduce demographic information such as gender and age. For instance, in the post: “Hi, I am a 19 y/o male. 6’2” and approximately 210 lbs. I currently take venlafaxine (Effexor) to treat depression and generalized anxiety disorder. I do not smoke. For the last several months, I have been experiencing hiccups…,” we inferred the demographic information as “19 y/o male.” (For a sample dataset, refer to Multimedia Appendix 1.)

This process constructed 36,252 posts with both inferred gender and age information. Preprocessing steps included text standardization such as lowercasing and stopwords removal. This dataset included 72.33% (25,972/36,252) of females and 27.67% (9936/36,252) of males, with 78% (28,277/36,252) of those aged 18‐35 years, reflecting a predominantly younger demographic.

**Table 1.** Sample AskaPatient dataset.

Name	Reason	Satisfaction	Side effects	Comments	Sex	Age (years)
Celexa	Anxiety and depression	4	Loss of appetite, headache, insomnia, and feeling numb	Definitely changing my life. I feel like a new person and ready to live every day like I never did. The side effects are not that bad, my body is slowly feeling normal again. Hoping not having any side effects soon!	Female	21
Celexa	Autism and anxiety	3	Increased aggression with higher doses (5 mg)	Took edge off my son’s anxiety but didn’t do much else. He was more aggressive when the dose was raised.	Male	9
Celexa	Fibromyalgia and depression	3	Weight gain and fatigue not helping with the pain from fibromyalgia	Quit taking feel like there is something that will work better. Having anger issues and uncontrollable outburst.	Female	56
Celexa	Anxiety	1	Hair loss, vision haze, heart palps, and laziness	This was the start of my downfall I lost all my hair from it and lost my life slowly avoid this crap as its led me down a path of destruction.	Male	24

### Analytical Techniques

To gain insights into patient experiences with antidepressants, we applied several advanced analytical methods, offering multiple perspectives on sentiment, emotions, personality traits, and latent themes.

#### Sentimental Analysis

We used the *twitter-roberta-base-sentiment-latest* model to classify text into negative, neutral, or positive sentiment. This model was trained on 124 million tweets from 2018 to 2021 and fine-tuned using the TweetEval benchmark [40]. For example, the comment “The side effects are not that bad, my body is slowly feeling normal again” yielded probabilities of 0.67 for positive, 0.28 for neutral, and 0.04 for negative sentiment. A 0.7 threshold was used for overall sentiment classification, but the full probability distributions were also analyzed for more nuanced insights.

#### Emotional Analysis

We used the English DistilRoBERTa-base model [41], optimized for detecting 6 basic emotions—anger, disgust, fear, joy, sadness, and surprise—along with neutral. This model trained on diverse datasets and enabled the identification of emotional states influencing antidepressant use.

#### Personality Analysis

LLMs, such as GPT-4 [42], were used for personality predictions, focusing on the Big Five traits—openness, conscientiousness, extraversion, agreeableness, and neuroticism. Each comment was assigned personality scores (1-5). Role prompting [43], a powerful artificial intelligence technique, was applied to guide the LLMs’ response in interpreting personality traits based on patient comments. (Detailed prompts can be found in Multimedia Appendix 1.)

#### Topic Modeling Insights

We used BERTopic [44], a topic modeling approach leveraging transformers and class-based term frequency-inverse document frequency to generate coherent topics. To enhance interpretability, we used GPT-4 to refine topic labels by analyzing keywords and representative documents. The following prompt was used (Textbox 1).

To further explore the themes of nonadherence and PCC within the generated topics, we selected the 2 predominate topics related to nonadherence (side effects, withdrawal symptoms, etc) and PCC (personalized treatment, communication with providers, etc). These topics were most populated, which allowed the study to focus on the exploration of how sentiment, emotion, and personality traits associated with the 2 themes.

Textbox 1. **Large language model prompt for topic label generation.**

Prompt = """

I have topic that contains the following documents: \n[DOCUMENTS]

The topic is described by the following keywords: [KEYWORDS]

Based on the above information, can you give a short label of the topic?

"""

### Ethical Considerations

The authors confirm that this research adhered to ethical guidelines and legal requirements in the United States. For the data obtained from AskaPatient, a data use agreement was signed to ensure compliance with ethical standards. Reddit data were sourced from publicly available platforms [45] and did not contain any personally identifiable information. The dataset was fully anonymized, and no private identifiers were included.

The data used in this research were obtained from 2 publicly accessible sources: AskaPatient and Reddit. Access to AskaPatient data was granted under a signed terms of use agreement between Consumer Health Resource Group, LLC, and authors, which provides a nonexclusive, revocable, and limited license for academic purposes. This agreement ensures compliance with ethical and legal requirements, including the stipulation that no more than 10 individual records or postings are included in any publication, with proper attribution to the AskaPatient website. Data from Reddit were publicly available and did not include any private, sensitive, or identifiable information. This study was partially supported by the National Science Foundation project (IIS-2041065) and adhered to the ethical guidelines and legal requirements outlined in the institutional review board regulations of Kent State University (reference no. KSU IRB20-182) and was classified as “exempt” under 45 CFR §46.104 (d), the federal policy for the protection of human subjects in the United States.

## Results

In our analysis, traditional statistical methods (eg, *t* tests) were used to validate the observed trends in sentiment and emotion across demographic groups, with detailed results provided in Multimedia Appendix 1.

### Sentiment Analysis

Significant gender differences were found in sentiment scores (Figures S4-S6 in Multimedia Appendix 1). Negative sentiment was higher in males (*t*_35,905_=−6.79; *P*<.001), while females exhibited higher neutral (*t*_35,905_=4.47; *P*<.001) and positive sentiment (*t*_35,905_=5.60; *P*<.001). The majority of posts were classified as neutral (21,499/36,253, 59.3%), followed by negative (13,922/36,253, 38.4%), and a smaller proportion of positive posts ( 798/36,253, 2.2%).

The gender difference in overall sentiment indicates that females exhibited 38.8% (10,181/26,245) negative, 59.1% (15,515/26,245) neutral, and 2.1% (551/26,245) positive sentiments, while males showed 37.6% (3764/10,008) negative, 59.8% (5986/10,008) neutral, and 2.6% (260/10,008) positive sentiments.

Across age groups, negative sentiment was notably higher in younger age groups (0‐18 years), while neutral sentiment was lower in the 0‐18 years age groups than in the older age groups, particularly 31‐45 years age group (*t*_35,903_=−15.77; *P*<.001). Positive sentiment was also significantly lower in younger age groups (0‐18 years) than in older age groups.

Sentiment by age range (Figure S7 in Multimedia Appendix 1) indicated that the negative sentiment was highest in the 0‐18 years age group (median score ~0.62) and gradually decreases with age, stabilizing around 0.52 for the 46‐60 and 60+ years age groups. Neutral sentiment was relatively stable across age, with the highest scores in the 31‐45 and 60+ years age groups (~0.37) and the lowest scores in the 0‐18 years age group (~0.31). Positive sentiment remained consistently low across all ages, peaking in the 46‐60 years age group (~0.14).

### Emotional Analysis

Significant gender differences were found for anger, disgust, joy, neutral, and sadness (*P*<.05). No significant differences were observed for fear and surprise between genders (Figures S10-S12 in Multimedia Appendix 1). The emotion analysis shows that fear was the most prevalent emotion across the dataset, present in 44.66% (16,196/36,253) of posts, followed by sadness (12,507/36,253, 34.49%). Surprise (2455/36,253, 6.77%) and anger (2132/36,253, 5.88%) were less common, while positive emotions such as joy appeared in only 4.88% (1769/36,253) of posts. Neutral emotions accounted for 2.67% (968/36,253), with disgust being the least frequent emotion (232/36,253, 0.64%).

A comparison of the mean emotion scores between females (**F**) and males (**M**) showed that fear was dominant for both genders (~0.40), with sadness slightly higher in females and joy more expressed in males, although both emotions had low overall score. Anger, disgust, and surprise were reported at similarly low levels for both genders, with females showing slightly higher levels of sadness and anger.

Across different age groups (0‐18, 19‐30, 31‐45, 46‐60, and 60+ years), fear remained the most prevalent emotion, peaking in the younger users (0‐18 years) and gradually declining with age. Sadness followed a similar pattern, with higher scores among younger users before decreasing. Joy and neutral emotions remain low across all age groups, with slight increases in users aged 46 years and older. Anger, disgust, and surprise showed no significant variation across age ranges.

### Personality Analysis

A gender difference was observed in agreeableness (*t*_35,903_=3.78; *P*<.001). No significant gender differences were found for openness, conscientiousness, extraversion, or neuroticism (Figures S1-S17 in Multimedia Appendix 1). The proportion of users who scored the highest level (5) across the 5 personality traits, separated by gender, showed that agreeableness was highest in males (429/10,008, 4.29%) compared with females (1034/26,24, 53.94%). Conscientiousness and extraversion showed similar proportions, with 4% (1039/25,972) of females and 4.06% (403/9936) of males scoring 5. Neuroticism exhibited minimal differences, with females at 3.97% (1031/25,972) and males at 4.05% (402/9936). Openness scores were also closely matched, with females scoring 4.07% (1056/25,972) and males scoring 3.93% (391/9,936).

Pairwise *t* tests across age groups for openness, conscientiousness, extraversion, agreeableness, and neuroticism showed no significant differences after adjustment (all *P*>.05). Personality trait scores varied across age group. Agreeableness showed little variation across age groups, peaking in the 60+ years age group (1511/36,253, 4.17%) and reaching its lowest in the younger than 30 years age group (1450/36,253, 4%). Conscientiousness remained stable, with slightly higher scores in the 19‐30 and 46‐60 years age groups (1462/36,253, 4.03%) and lower scores in the 60+ years age group (1355/36,253, 3.74%). Extraversion was highest in the 60+ years age group (1555/36,253, 4.29%) and lowest in the 0‐18+ years age group (1348/36,253, 3.72%). Neuroticism peaked in the 60+ years age group (1627/36,253, 4.49%) and was lowest in the 46‐60 years age group (1360/36,253, 3.75%). Openness followed a similar trend, with the 60+ years age group scoring the highest (1704/36,253, 4.70%) and the 46‐60 years age group scoring the lowest (1414/36,253, 3.90%).

### Topic Analysis

Table 2 shows the results of BERTopic analysis within dataset, identifying key topics related to antidepressants user experiences. The largest topic, “Mental Health and Relationships,” accounted for 11.69% (3755/32,121) of the discussions, indicating a significant focus on managing these mental health conditions. Other notable topics included “Lexapro Experiences and Alternatives” (2292/32,121, 7.14%) and “Zoloft Withdrawal and Anxiety” (2184/32,121, 6.80%), reflecting common concerns about antidepressant experience and desire to switch to alternative medicines to manage their anxiety and depression.

Several discussions centered around side effects, including “Medication and Sexual Dysfunction” (2146/32,121, 6.68%) , “Chronic Abdominal Pain Issues (1949/32,121, 6.07%), “Skin Rashes and Sensitivities” (1700/32,121, 5.70%), “Heart Symptoms and Anxiety” (1638/32,121, 5.10%), and “Chronic Pain and Health Issues” (1300/32,121, 4.05%), alongside concerns related to weight gain and sleep disorders, which highlight frequent side effects users encounter during treatment.

Two primary themes—nonadherence and self-care—were extracted from the dataset based on the highest documented counts from relevant topics identified by the BERTopic model. In the nonadherence theme, “Zoloft Withdrawal and Anxiety,” ” Effexor Experiences and Withdrawals,” “Cymbalta Withdrawal Experiences and Effects,” and “Psychiatric Drug Withdrawal Effects” were assigned. In the self-care theme, the topics “mental health and relationships,” “relationship struggles and mental health,” and “toxic relationships and mental health” were chosen. Furthermore, we investigate sentiment, emotion, and personality traits across the 2 themes.

Sentiment distribution varied between the themes of nonadherence and self-care (Figure S19 in Multimedia Appendix 1). Neutral sentiment was most prevalent in both, accounting for 57.70% (3679/6376) in nonadherence and 47.65% (2929/6147) in self-care. Negative sentiment followed closely, with 36.68% (2339/6376) in nonadherence and 50.69% (3116/6147) in self-care, while positive sentiment remained low (358/6376, 5.61% and 102/6147, 1.66%, respectively).

The emotion distribution has been compared across the themes of nonadherence and self-care (Figure S20 in Multimedia Appendix 1). Fear was dominant emotion in both themes, making up 55.83% (3560/6376) of emotions in nonadherence and 35.90% (2207/6147) in self-care. Sadness was more prominent in self-care (2733/6147, 44.46%) than in nonadherence (1566/6376, 24.56%). Anger was also more frequent in self-care (592/6147, 9.63%) compared with nonadherence (265/6376, 4.16%).

Personality traits (scoring 5) also differed across themes (Figure S21 in Multimedia Appendix 1). In the nonadherence theme, agreeableness was the most common trait (2023/6376, 31.73% ), followed by extraversion (1983/6376, 31.10%) and neuroticism (1892/6376, 29.68%). In the self-care theme, neuroticism was highest (1891/6147, 30.76%), followed by openness (1887/6147, 30.69%), conscientiousness (1804/6147, 29.34%), and agreeableness (1789/6,147, 29.10%).

**Table 2.** BERTopic result.

Topic	OpenAI label	Document count	Proportion
0	Mental Health and Relationships	3755	11.69
1	Lexapro Experiences and Alternatives	2292	7.14
2	Zoloft Withdrawal and Anxiety	2184	6.80
3	Medication and Sexual Dysfunction	2146	6.68
4	ADHD^a^ Medication Challenges and Insights	2090	6.51
5	Chronic Abdominal Pain Issues	1949	6.07
6	Skin Rashes and Sensitivities	1700	5.70
7	Chronic Pain and Fatigue Issues	1651	5.14
8	Heart Symptoms and Anxiety	1638	5.10
9	Abnormal Blood Test Concerns	1446	4.50
10	Chronic Pain and Health Issues	1300	4.05
11	Weight Gain and Medication Struggles	1272	3.96
12	Relationship Struggles and Mental Health	1260	3.92
13	Experiences With Prozac for Anxiety	1176	3.66
14	Irregular Periods and Birth Control	1146	3.57
15	Toxic Relationships and Mental Health	1132	3.53
16	Wellbutrin Experiences and Challenges	1006	3.13
17	Chronic Sleep Disorders and Fatigue	990	3.08
18	Dizziness and Anxiety Symptoms	788	2.45
19	Chronic Headache and Migraine	773	2.40
20	Psychiatric Drug Withdrawal Effects	766	2.38
21	Anxiety and Substance Reactions	734	2.28
22	Effexor Experiences and Withdrawals	681	2.12
23	Chronic UTI^b^ Symptoms and Challenges	517	1.61
24	Cymbalta Withdrawal Experiences and Effects	453	1.41
25	Alcohol, Anxiety, and Treatment Concerns	402	1.25
27	Eye Health and Vision Concerns	385	1.20
28	Chronic Ear Infections and Tinnitus	274	0.85
29	Managing Anxiety and Depression Tips	2	0.01

a ADHD: attention-deficit/hyperactivity disorder.

b UTI: urinary tract infection.

## Discussion

### Principal Findings

The finding reveals that sentiment of most of the antidepressant users is neutral within the dataset, while there remains substantial negative sentiment, and few show positive. In the meantime, the emotional analysis exhibits that fear and sadness command patients’ emotional response. Those findings may consider the experiences when the patients use those antidepressants during the daily life. The community and health care can provide support to reduce the fear of side effects or failure of antidepressants, and family can facilitate comfort toward the sadness and negative emotions. Consequently, antidepressant users can practice self-care and enhance their treatment adherence.

Personality has not indicated a significant difference within the dataset. Topic analysis expresses that discussions mainly emphasize managing anxiety and depression, the efficacy of medications, and their side effects. Furthermore, in the nonadherence theme, the neutral sentiment was more prominent, followed by the significant fear emotion. In the self-care theme, the negative sentiment was more distinct, with the sadness emotion most dominant. Those findings suggest that the most beneficial route for antidepressant users is to exercise focused self-care for their mental health issues, while continuing their antidepressants treatment.

### RQ1: How Do Sentiment Analyses Reveal Differences in Antidepressant Experiences Across Demographics?

The sentiment analysis indicates that public perception of antidepressants is predominantly neutral, with a substantial portion reflecting negative sentiment and very few positive expressions. The neutral sentiment suggests ambivalence or cautious optimism toward antidepressants, while the limited positive sentiment points to restricted satisfaction with treatment outcomes. These trends reflect broader society uncertainty or skepticism about antidepressant effectiveness and side effects, consistent with the literature identifying nonadherence as a pervasive issue [46].

In terms of gender, the results show minor differences in sentiment between males and females, with females exhibiting slightly higher negative sentiment and lower positive sentiment than males. This may reflect the differences in antidepressant experiences, societal pressures, or varying expectations regarding treatment outcomes across genders. Addressing these gender-specific concerns may enhance treatment satisfaction and adherence of antidepressants.

Notably, younger individuals expressed the highest levels of negative sentiment, which gradually decreases with age. This could be attributed to heightened sensitivity to side effects or unaccomplished expectations from treatment among younger users [47]. Older age groups demonstrated more stable neutral sentiment and slightly higher positive sentiment, particularly in the 46‐60 years age group. These findings underscore the importance of patient’s sentiment during antidepressant treatment. Mental health may benefit from tailoring interventions based on these emotional responses. Therefore, addressing negative bias early may be a key mechanism of antidepressant drug action and a potentially useful predictor of therapeutic response [48].

### RQ2: What Predominant Emotions (eg, Fear, Sadness, and Joy) Are Associated With Antidepressant Use, and How Do These Vary by Demographics?

The emotional analysis revealed that fear and sadness dominate patients’ emotional responses, with fear accounting for nearly half (44.66%) and sadness is the second most prominent emotion (34.49%). These findings suggest that patients’ experiences with antidepressants are characterized by deep concerns and dissatisfaction. The prominence of fear, especially among younger users, may indicate anxiety related to side effects, potential long-term dependence, or uncertainties regarding the treatment’s efficacy. This aligns with research showing that emotional blunting, commonly associated with antidepressants use, is also a symptom of depression and is linked to poorer remission outcomes [49].

The prominence of fear across both genders highlights widespread concerns about side effects, dependence, and treatment efficacy. However, the slightly higher level of sadness and anger in females suggests gender-specific emotional responses to antidepressants. Tailored communication strategies that address these gender-specific emotional concerns could improve treatment satisfaction and adherence.

As patients age, fear tends to decrease, potentially due to greater familiarity with antidepressants or prolonged exposure to treatment. However, the emotional burden remains strong among younger users, emphasizing the need for enhanced communication regarding treatment plans and potential side effects. The high prevalence of sadness**,** particularly among younger users, may reflect frustration or disappointment with treatment outcomes, potentially linked to unmet expectations, a lack of education over antidepressant, or emotional blunting. The intent of providing time-phased educational materials to patients is to maximize the relevance of such information by synchronizing it with typical recovery processes and issues. Additional efforts at engaging patients earlier after the initiation of treatment might be of most benefit [50].

### RQ3: What Personality Traits (eg, Extraversion and Neuroticism) Are Prevalent Among Users Discussing Antidepressants, and How Do These Traits Differ Across Demographics?

Personality traits exhibited minor yet meaningful variations across gender and age groups. Agreeableness was higher in males, suggesting that empathy and social cooperation may positively influence adherence to mental health treatment. Concerning the age difference, one study found that the lower the extroversion score, the better the response to antidepressant treatment for adolescents [51]. From our finding, extraversion was highest in the 60+ years age group (4.29%) and lowest in the 0‐18 years age group (3.72%). Compared with the higher negative sentiment, fear, and sadness emotions among the 0‐18 years age group, the health care providers may tailor treatment to maximize the outcome of antidepressant medication.

Agreeableness is dominant in the nonadherence theme; neuroticism is the largest personality in the self-care theme. These are not notable findings that align with existing literatures for personality impact to antidepressant adherence and self-care; the result may provide a new perspective in the future research.

### RQ4: What Are the Prevailing Topics in Web-Based Antidepressant Discussions, and How Do Sentiment, Emotion, and Personality Insights Inform Our Understanding of Nonadherence and Self-Care Themes?

The topic analysis identified that discussions primarily focus on mental health and relationships, medication efficacy, and side effects such as sexual dysfunction and withdrawal symptoms. These findings highlight clear concerns among users regarding the mental health issues and overall effectiveness of antidepressants. The dominance of neutral and negative sentiment in these discussions, combined with the high presence of fear and sadness, indicates that many users remain uncertain about their treatment outcomes and dissatisfy their daily life regarding their experience with the antidepressants.

Discussions around nonadherence are marked by fear, followed by sadness emotion, with patients expressing concerns about withdrawal symptoms and medication efficacy. In contrast, self-care discussions show a notable trend at sadness, accompanied by an increase in expressions of anger. A study found that stigma and fear of adverse events inhibited initiation of therapy, while adverse events and ineffectiveness of antidepressants contributed to discontinuation. Patients with strong perceptions of the necessity and few concerns about antidepressants were more likely to adhere to treatment at all phases of adherence [52].

### Limitations

This study has several limitations. First, it relies on self-reported data from AskaPatient and Reddit, which may introduce bias as individuals who share their experiences on the web may not represent the broader population of antidepressant users. These users may disproportionately report extreme experiences, potentially skewing the findings. Furthermore, the focus on social media users limits the generalizability of the results to the wider population.

Although sentiment and emotion analysis tools are sophisticated, they may not fully capture the complexity of human emotions, particularly mixed or nuanced sentiments. Personality traits inferred from text via LLMs might not always align with real-world behaviors, possibly leading to misinterpretations. Furthermore, the study provides a snapshot of discussions at a particular time, without tracking changes over time. Furthermore, while age and gender are considered in the analysis, other demographic factors, such as socioeconomic status, education, and geographic location, were not controlled for and may influence user experiences with antidepressants. These factors should be considered in future studies to provide a more comprehensive understanding of patients’ antidepressant use.

### Conclusions

This study offers valuable insights into public perceptions of antidepressants, revealing that neutral and negative sentiments dominate discussions, with fear and sadness as the most common emotions, particularly among younger users. Personality traits exhibited minor yet meaningful variations across gender and age groups. Agreeableness was higher in males, suggesting that empathy and social cooperation may positively influence adherence to mental health treatment.

The topic analysis revealed that discussions primarily revolve around mental health and relationships, medication efficacy, and side effects, such as sexual dysfunction and withdrawal symptoms. Discussions about nonadherence are characterized by fear as the dominant emotion, followed by sadness, with patients voicing concerns about withdrawal symptoms and the effectiveness of medications. In contrast, self-care discussions convey a predominant sense of sadness, accompanied by heightened expression of anger.

The findings underscore the importance of self-care that addresses psychological dimensions of antidepressant use. Tailored interventions based on individual negative sentiment, fear, and sadness emotional challenges could improve overall treatment outcomes. By leveraging real-time social media data and advanced analytical tools, this research contributes to a more patient-centered understanding of antidepressant use, offering pathways for improving patient care and enhancing their nonadherence for antidepressants.

## Supplementary material

10.2196/62680 Multimedia Appendix 1 Detailed supplementary data on public perceptions of antidepressants discussed on social media, including topic analysis results, sentiment breakdowns, and associated statistical summaries.

## Abbreviations

LLM: large language model
NLP: natural language processing
PCC: patient-centered care
RQ: research question
