# Supplementary material for: Probing Public Perceptions of Antidepressants on Social Media: Mixed Methods Study
Source: JMIR Form Res. 2025 Feb 26;9:e62680. doi: 10.2196/62680 (PMC11882120; doi:10.2196/62680)
Supplement: Multimedia Appendix 1 [file formative-v9-e62680-s001.docx]

# 1 Data and Methods

## Data Collection and Preprocessing for Antidepressant Analysis

**Table S1 Sample data of Reddit**

**
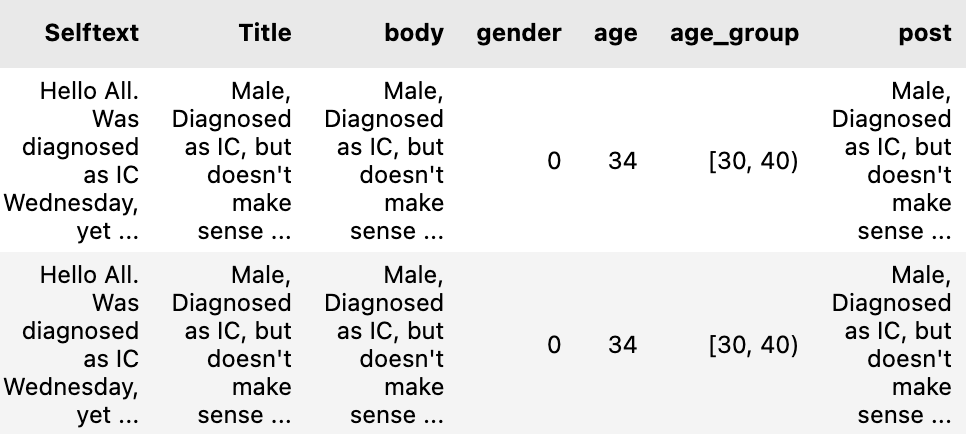
**


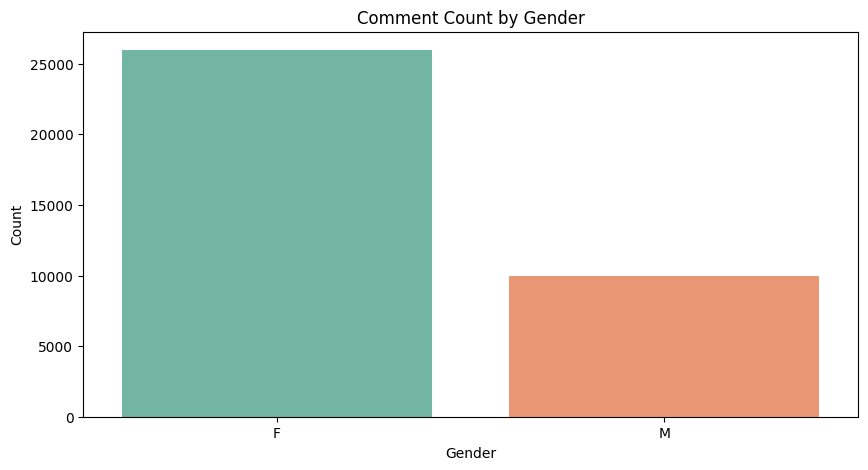


**Figure S1.** Data distribution by gender.

Figure 1 illustrates the distribution of comment counts by gender in the dataset. It reveals that female participants (F) have contributed significantly more comments than their male counterparts (M). Notably, the volume of comments made by females is more than double that of males.

**
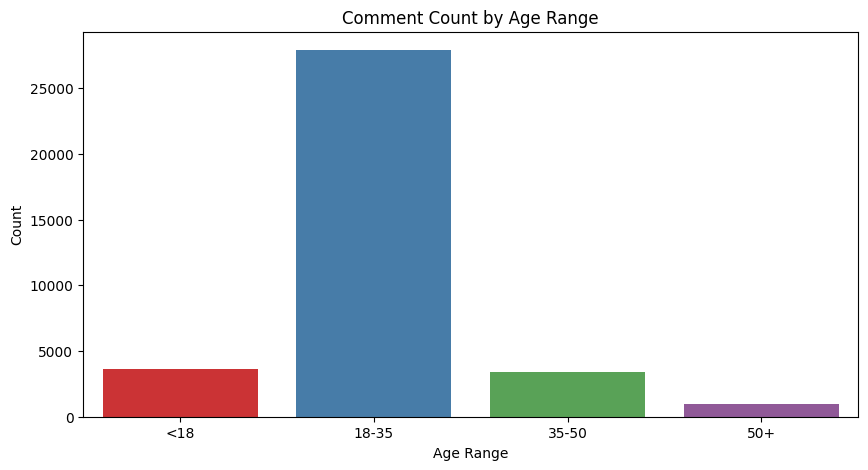
**

**Figure S2.** Data distribution by age range.

Figure 2 displays the distribution of comment counts segmented by age range. It is evident from the chart that the 18-35 age group is the most active, contributing a significantly higher number of comments compared to other age groups. In contrast, the least amount of activity is observed in the 50+ age group, with the <18 and 35-50 age groups also showing relatively low but more comparable activity levels.

**
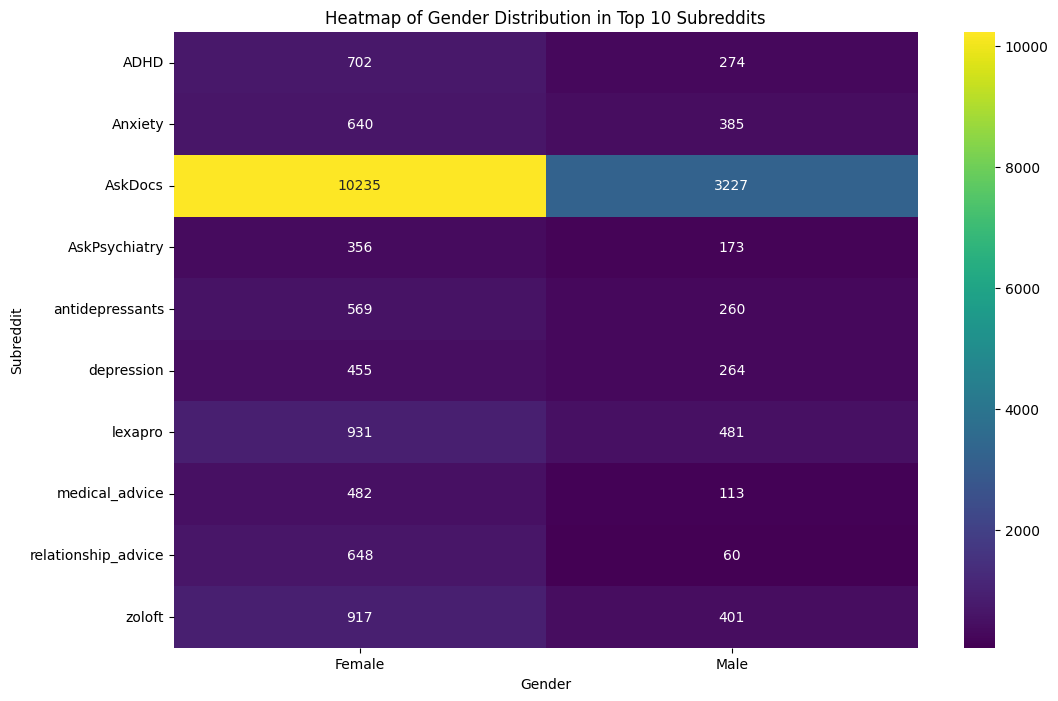
**

**Figure S3.** Subreddit distribution correlation with gender.

Figure 3 presents a heatmap illustrating gender distribution across the top 10 subreddits relevant to mental health and medical advice. This visual highlight a pronounced trend of higher female participation across most subreddits, especially notable in 'AskDocs' where female posts substantially outnumber male posts (10,235 females vs. 3,227 males). Other subreddits, including 'ADHD', 'Anxiety', 'depression', and 'lexapro', similarly show greater female engagement. The color gradient from purple to yellow indicates increasing comment counts, with 'AskDocs' not only leading in female participation but also as the most active subreddit overall in this dataset.

## Result

In our analysis, traditional statistical methods (e.g., t-tests) were used to validate the observed trends in sentiment and emotion across demographic groups.

### Sentiment Analysis


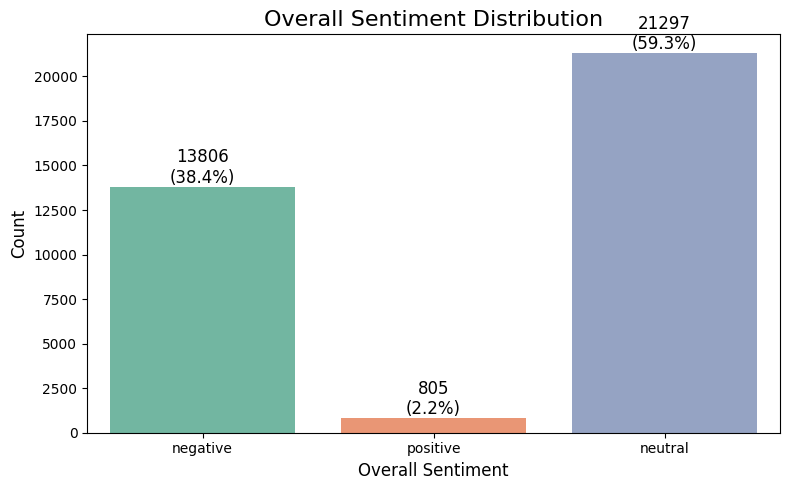


**Figure S4.** Overall sentiment distribution of user-generated posts about antidepressants.


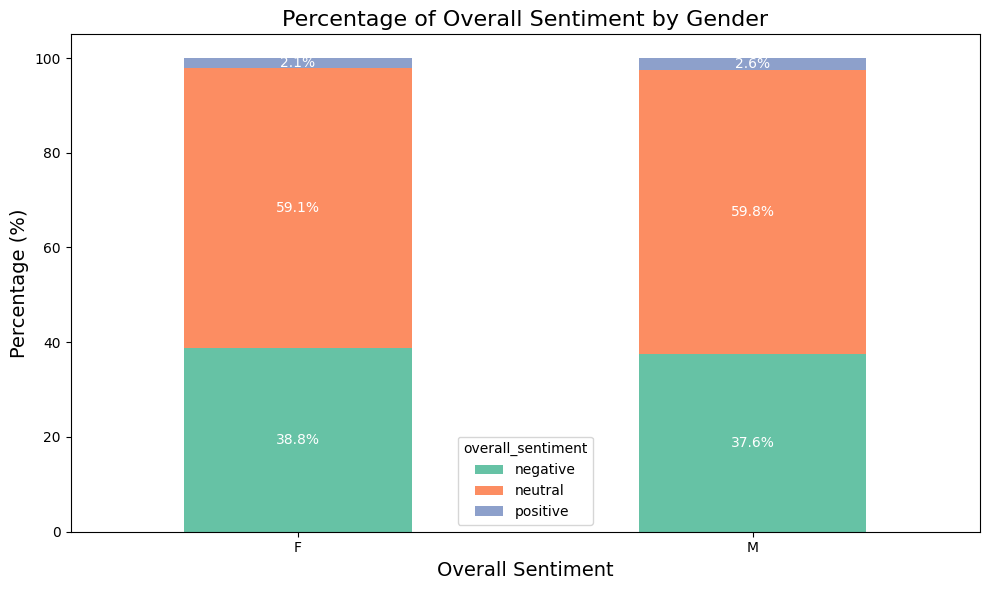


**Figure S5.** Overall sentiment by gender.


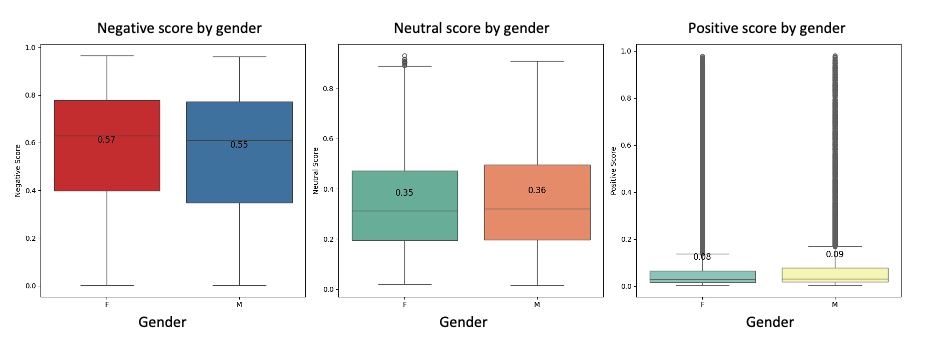


**Figure S6.** Sentiment score by gender.


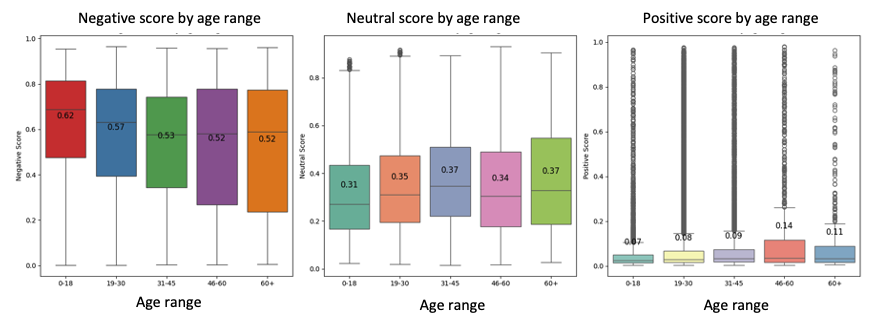


**Figure S7.** Sentiment scores by age range.

To assess the relationship between sentiment and demographic factors, we performed statistical analyses on gender and age. For gender, a t-test analysis revealed significant differences in emotional responses. Specifically, males exhibited more negative sentiment (t-statistic: -6.79, p = 1.15E-11), while females demonstrated higher levels of neutrality (t-statistic: 4.47, p = 7.78E-06) and positivity (t-statistic: 5.60, p = 2.11E-08). Additionally, a chi-square analysis was conducted to examine the relationship between sentiment and age. The relation between these variables was significant, χ² (2, N = 35,908) = 14.14, p < .01, indicating that age also plays a significant role in shaping emotional responses.

Table S2 T_test Result for Sentiment by Gender

| Sentiment Score | T-statistic | P-value | Significant |
| --- | --- | --- | --- |
| Negative Score | -6.79 | 1.15E-11 | + |
| Neutral Score | 4.47 | 7.78E-06 | + |
| Positive Score | 5.6 | 2.11E-08 | + |


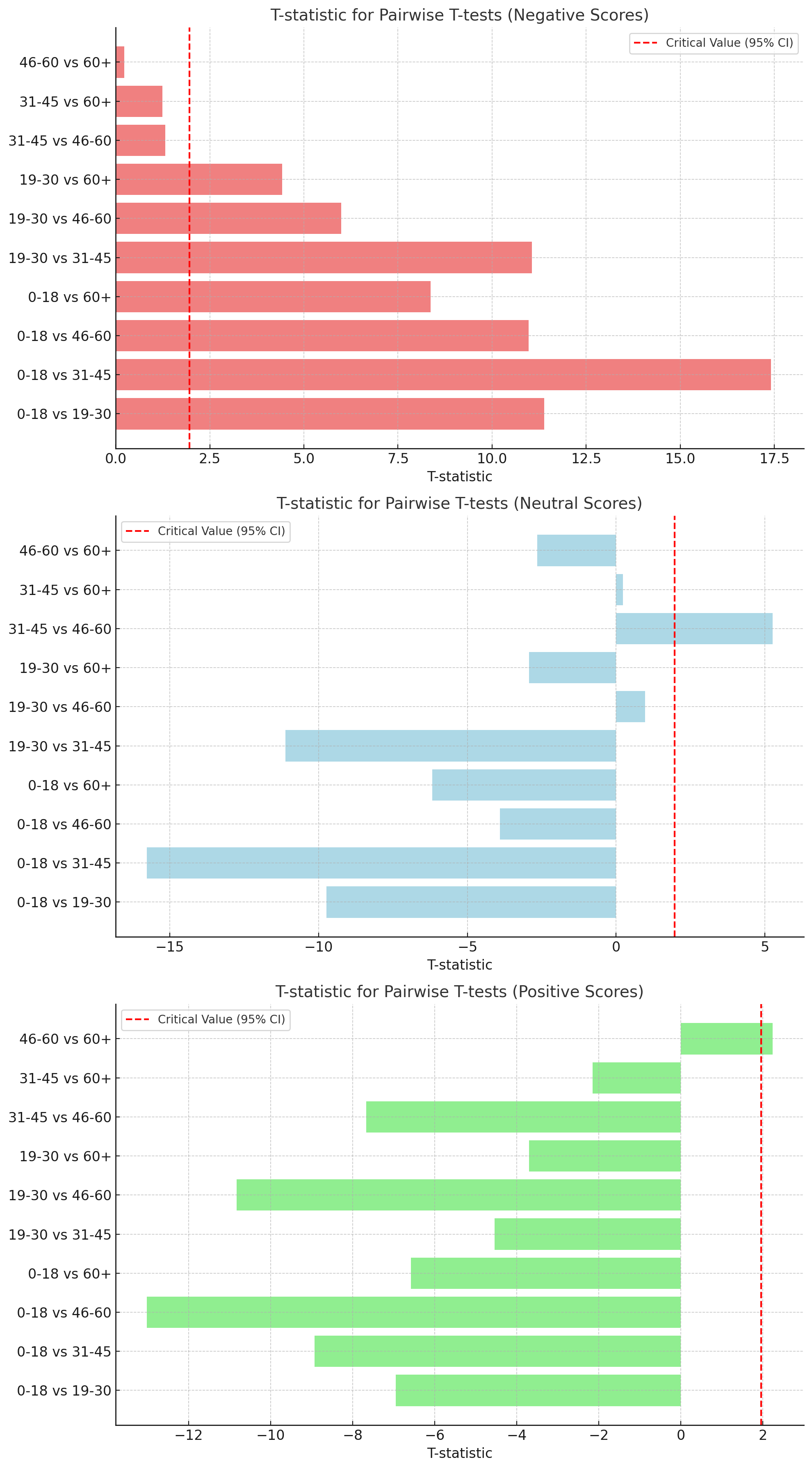


**Figure S8.** T-statistic for pairwise T-tests (sentiment score).

The pairwise t-tests reveal significant differences in negative, neutral, and positive sentiment scores across most age group comparisons. The strongest differences are observed between younger (0-18) and older age groups, particularly in negative and positive scores, with many t-statistics exceeding the critical threshold of 1.96, indicating statistical significance.

### Emotional Analysis

Table S3 T_test emotion results by gender

| Emotion | t_statistic | p_value | Significant |
| --- | --- | --- | --- |
| Anger | -5.494354 | 3.948026E-08 | + |
| Disgust | -2.884497 | 0.003922732 | + |
| Fear | 0.213283 | 0.8311072 | - |
| Joy | 9.909073 | 4.070551E-23 | + |
| Neutral | 7.52211 | 5.515616E-14 | + |
| Sadness | -3.093573 | 0.001979135 | + |
| Surprise | -1.579907 | 0.114137 | - |

To assess the relationship between emotions and gender, we performed statistical t-test analyses. For **anger**, the results revealed that males exhibited significantly higher levels (t-statistic: -5.49, p = 3.95E-08), while for **joy** (t-statistic: 9.91, p = 4.07E-23) and **neutral** emotion (t-statistic: 7.52, p = 5.52E-14), females demonstrated notably higher levels. Additionally, males showed higher levels of **sadness** (t-statistic: -3.09, p = 0.00198), and **disgust** also showed a significant difference (t-statistic: -2.88, p = 0.00392). However, no significant gender differences were found for **fear** (t-statistic: 0.21, p = 0.831) and **surprise** (t-statistic: -1.58, p = 0.114).


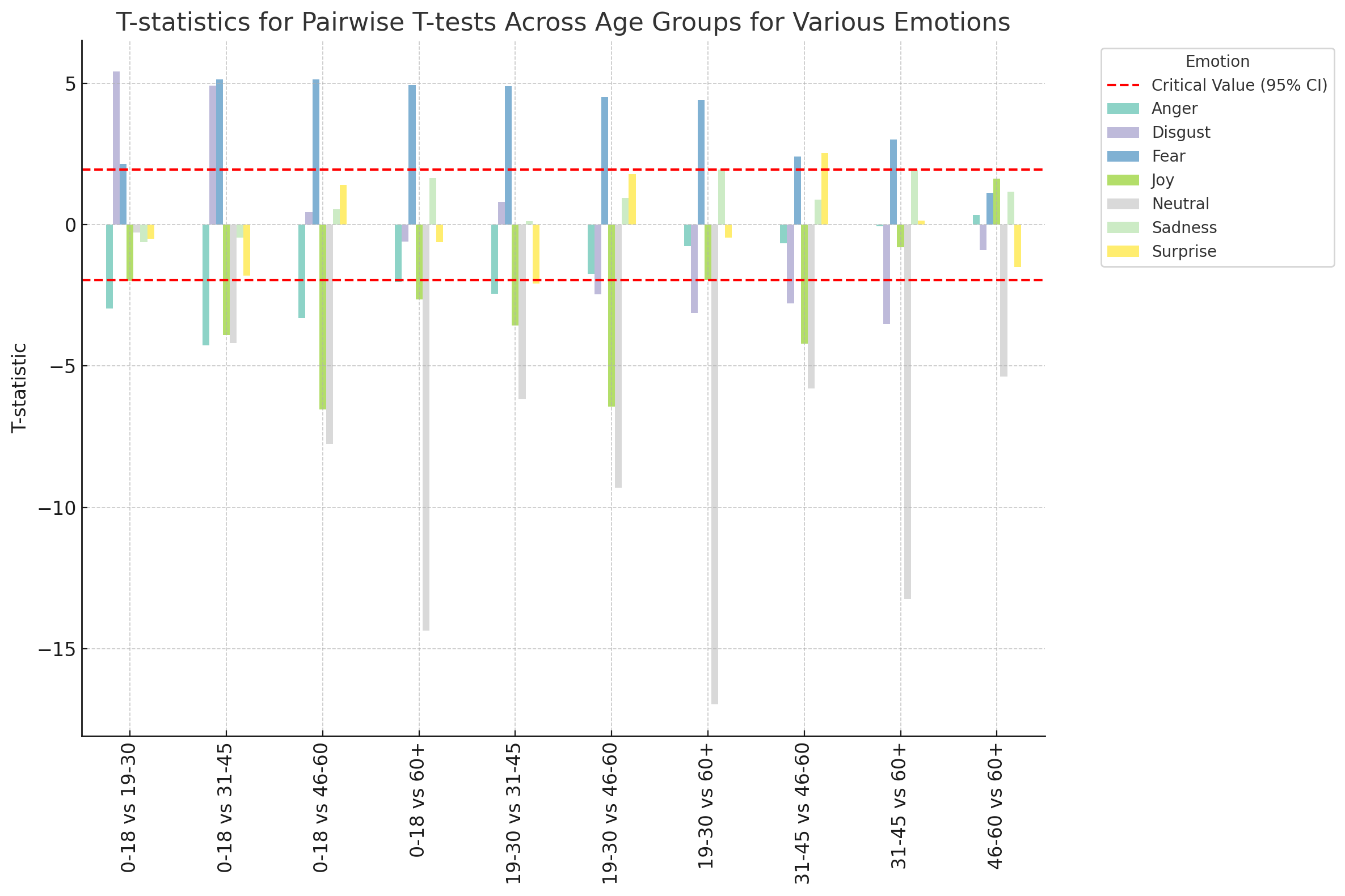


**Figure S9.** T-statistic for pairwise T-tests (emotion score).

The t-test analysis reveals significant emotional differences across several age groups. For example, fear shows large differences between 0-18 vs. 31-45 (t = 5.15) and 19-30 vs. 46-60 (t = 4.53), while disgust is notably different in 0-18 vs. 19-30 (t = 5.41) and 0-18 vs. 31-45 (t = 4.93). Significant variation in joy is seen between 0-18 vs. 46-60 (t = -6.54), and neutral emotions differ strongly in 19-30 vs. 60+ (t = -16.97). These findings highlight substantial emotional shifts, particularly between younger and older age groups.


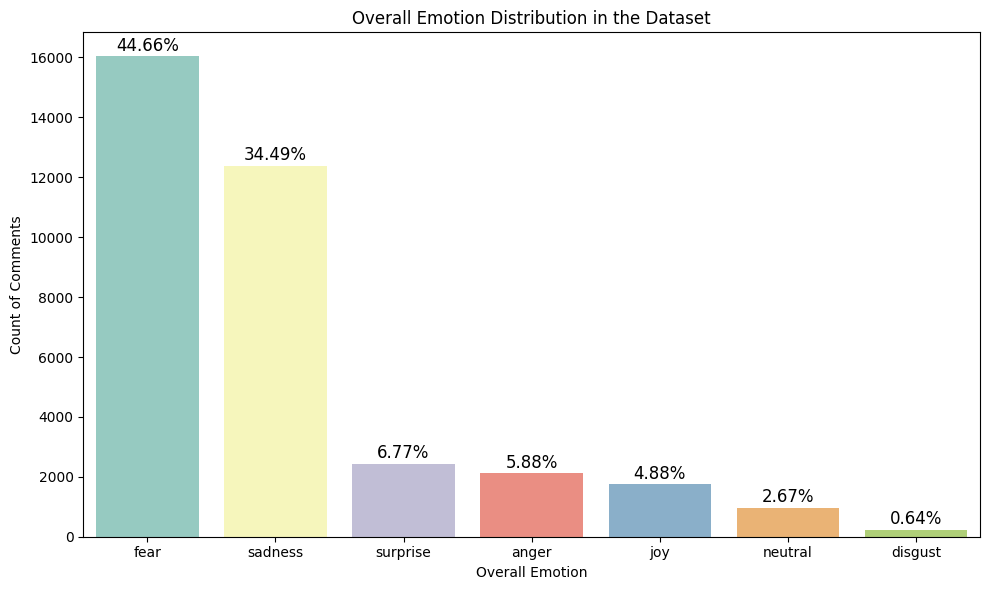


**Figure S10.** Overall emotion distribution.


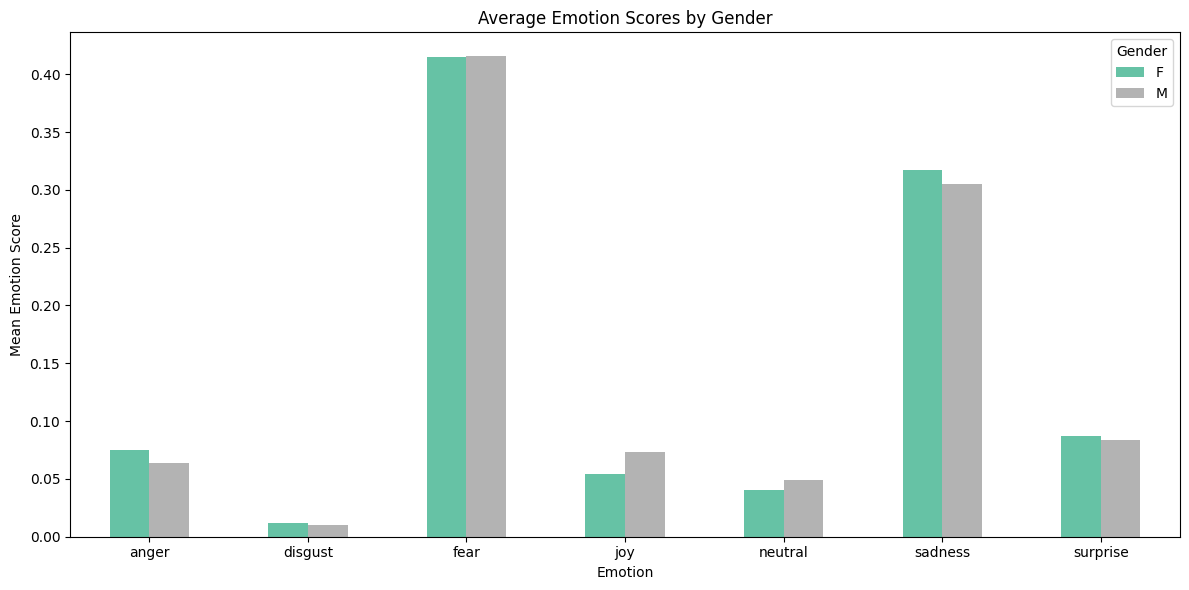


**Figure S11.** Average emotion scores by gender.


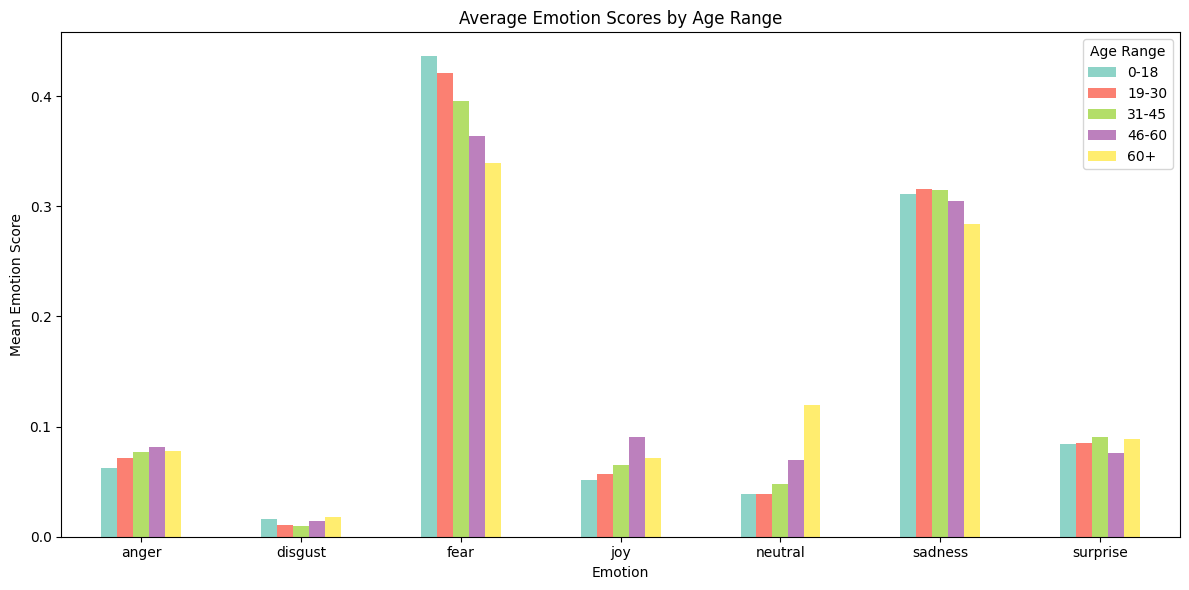


**Figure S12.** Average emotion scores by age range.

### Personality Analysis

Table S4 T_test personality results by gender

| Personality Trait | t_statistic | p_value | Significant |
| --- | --- | --- | --- |
| Openness | -0.410381 | 0.681529 | - |
| Conscientiousness | 1.009663 | 0.312664 | - |
| Extraversion | 0.755414 | 0.450006 | - |
| Agreeableness | 3.784074 | 0.000155 | + |
| Neuroticism | 0.490125 | 0.624048 | - |

The t-test results show no statistically significant gender differences across the five personality traits—Openness, Conscientiousness, Extraversion, Agreeableness, and Neuroticism—as none of the p-values are below 0.05.

The t-test results for personality features across genders indicate that only Agreeableness shows a significant difference (t-statistic: 3.78, p < 0.001), exceeding the critical threshold of ±1.96. Other personality traits, including Openness, Conscientiousness, Extraversion, and Neuroticism, have t-statistics within the non-significant range, indicating no substantial gender differences for these traits.


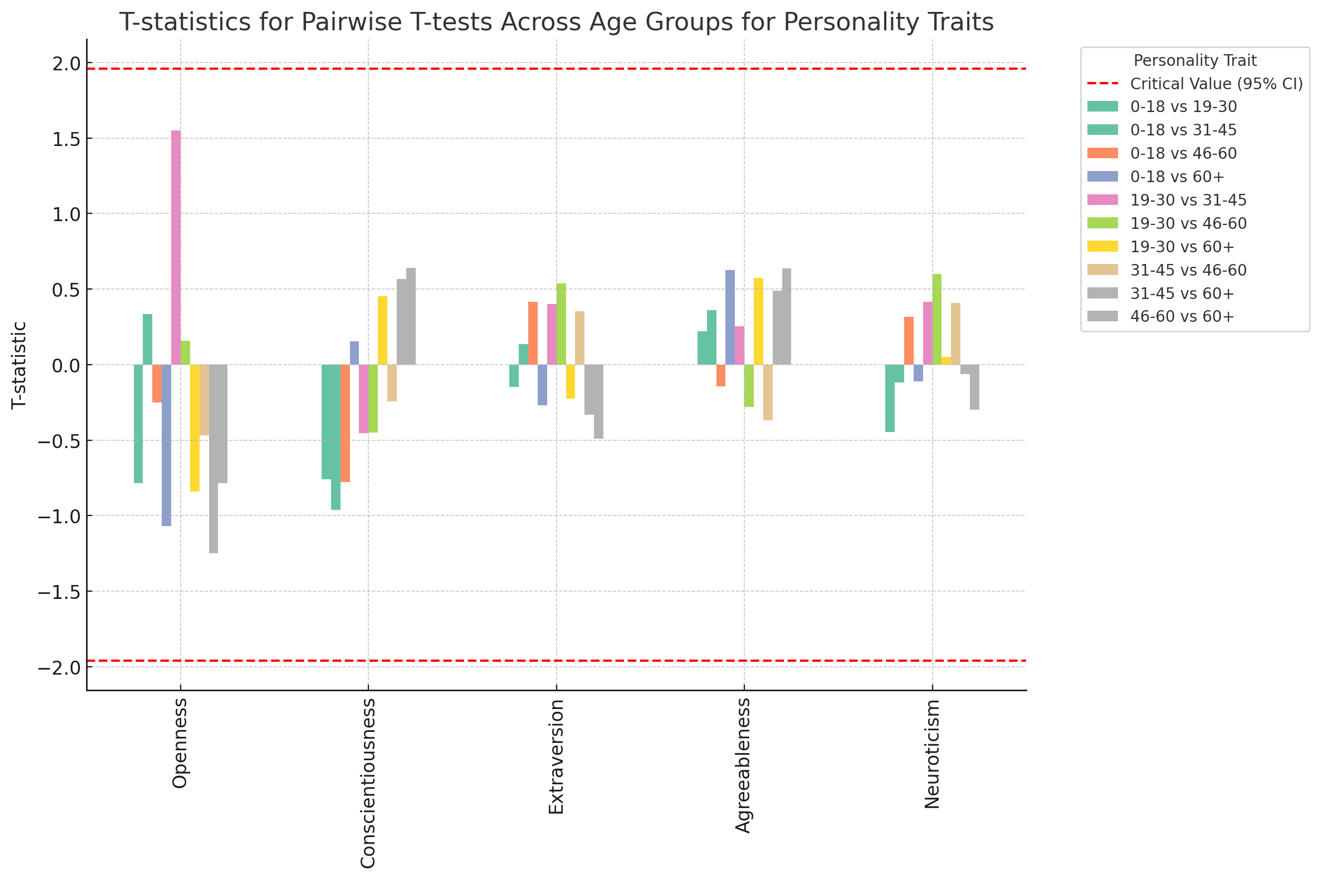


**Figure S13.** T-statistic for pairwise T-tests (personality score).

The t-test results for personality traits across age groups indicate no significant differences, as all t-statistics fall within the non-significant range (±1.96). This is true for Openness, Conscientiousness, Extraversion, Agreeableness, and Neuroticism. Although some comparisons, such as Openness between 19-30 and 31-45, show relatively larger t-statistics, they remain below the critical value, suggesting no statistically significant differences across age groups for these personality traits.

**
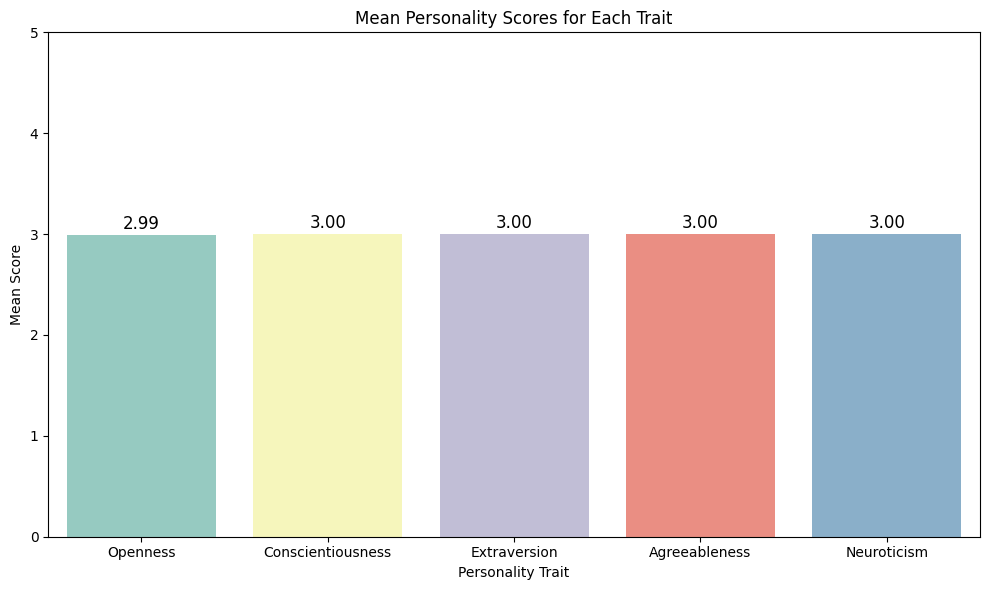
**

**Figure S14.** Mean personality scores.


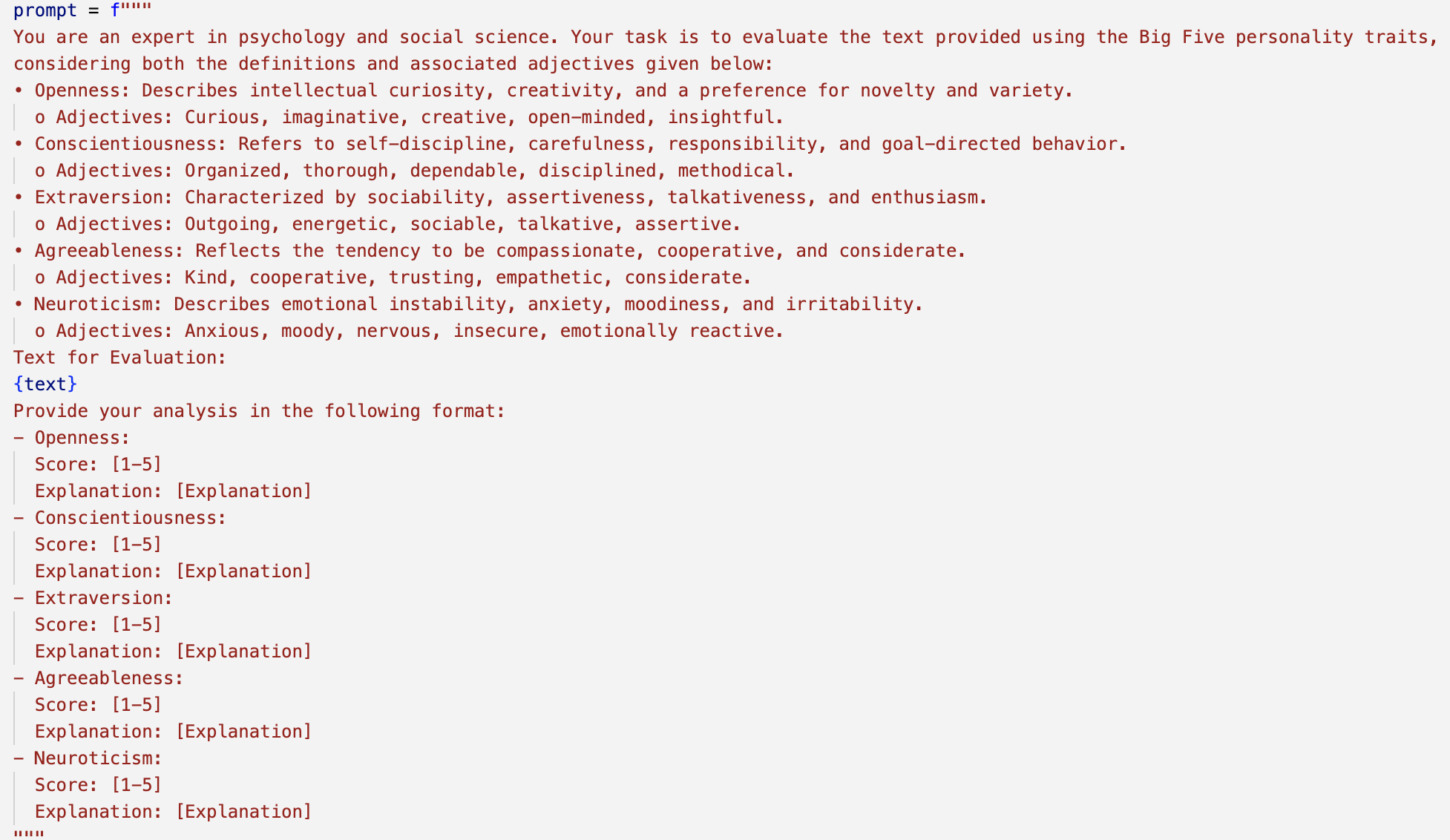


**Figure S15.** Personality prompt via LLMs.


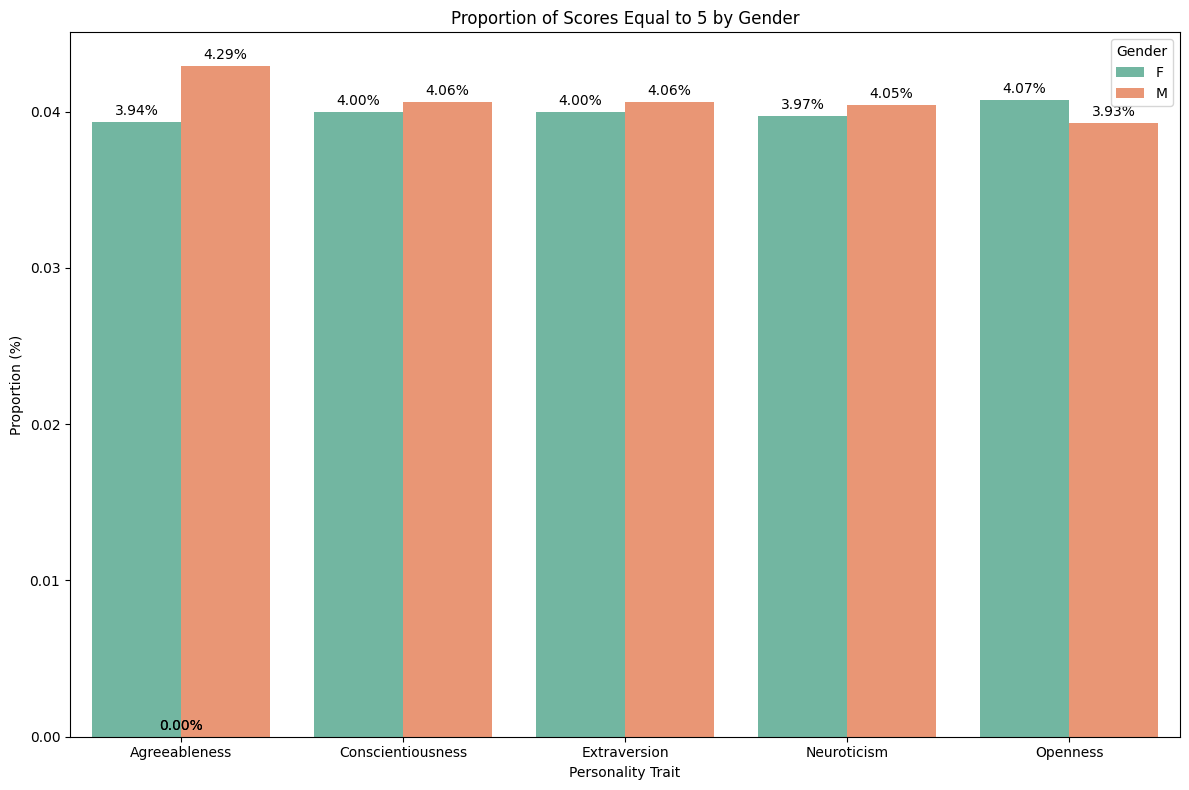


**Figure S16.** Proportion of scores equal to 5 by gender.


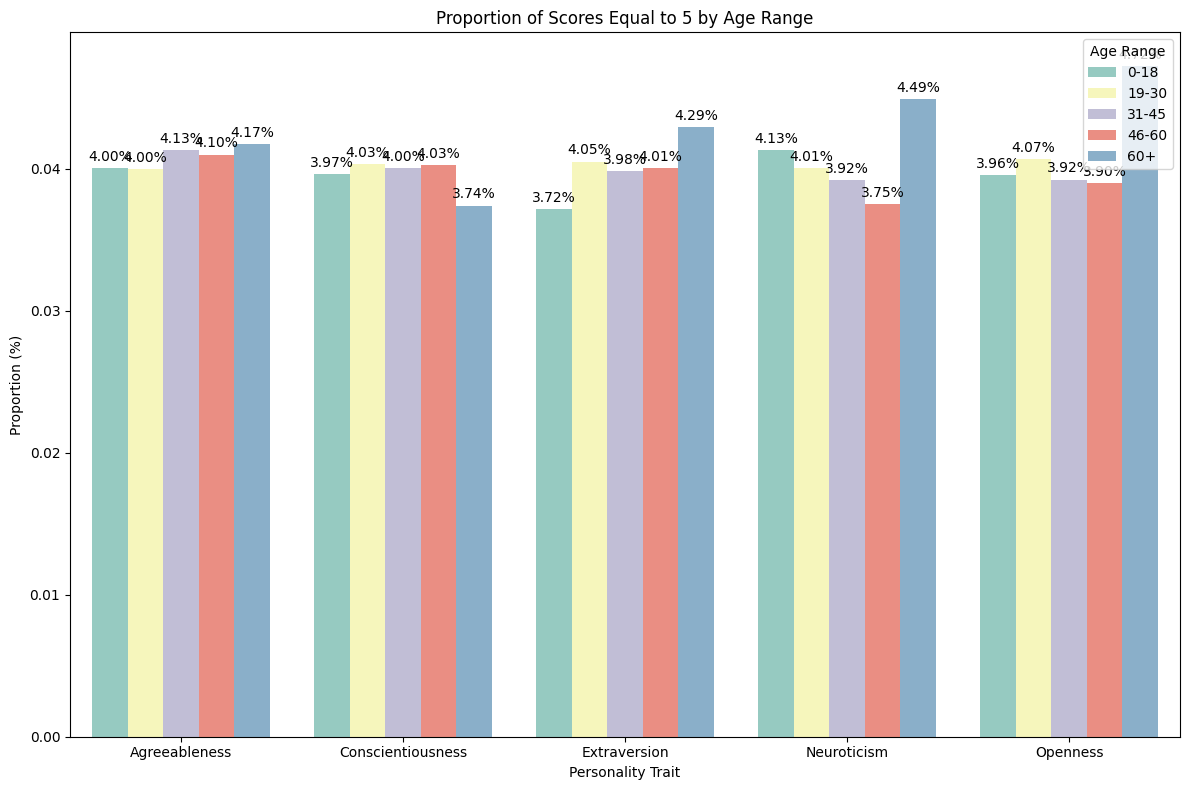


**Figure S17.** Proportion of scores equal to 5 by age range.

### Topic Analysis


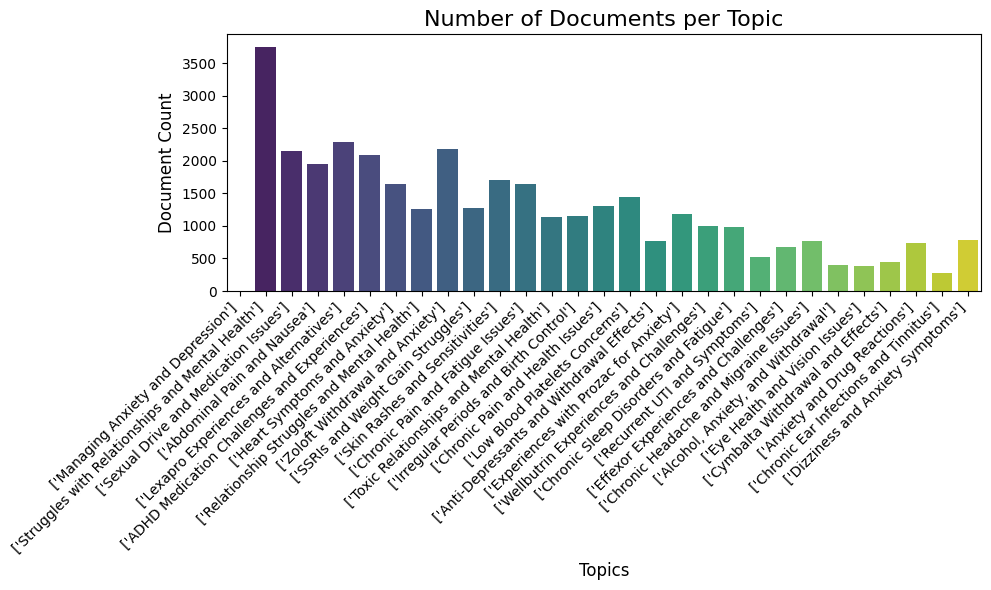


**Figure S18.** Topics distribution within data.


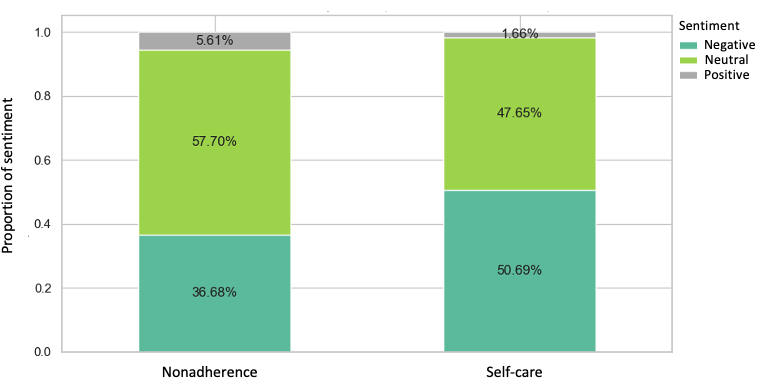


**Figure S19.** Sentiment distribution by theme (nonadherence vs. self-care).


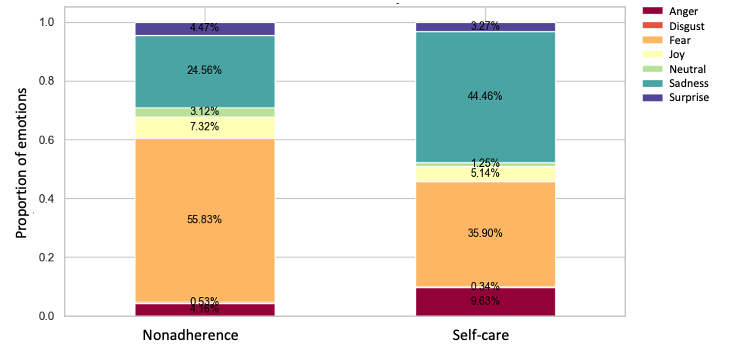


**Figure S20.** Emotion distribution by theme (nonadherence vs. self-care).


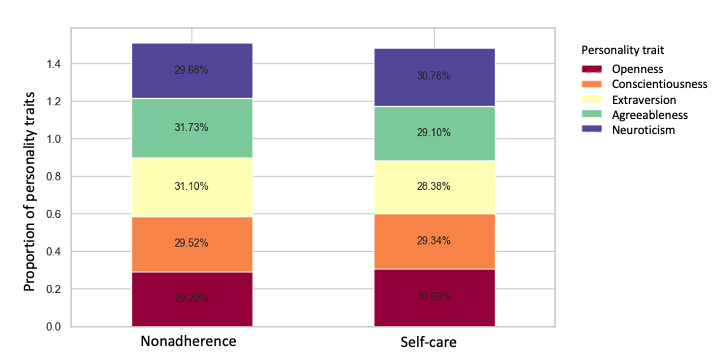


**Figure S21.** Personality traits (score = 5) distribution by theme (nonadherence vs self- care).
